# Supplementary material for: An open-source framework for large-scale, flexible evaluation of biomedical text mining systems
Source: J Biomed Discov Collab. 2008 Jan 29;3:1. doi: 10.1186/1747-5333-3-1 (PMC2276192; doi:10.1186/1747-5333-3-1)
Supplement: Additional file 1 — Evaluation platform source code. The additional file contains the software evaluation framework discussed in this paper. The most current release can be downloaded from SourceForge.net [1]. See the accompanying README file for instructions on installation and use. Also included in the distribution is a collection of UIMA wrappers for some commonly used BioNLP tools and annotated corpora. [file 1747-5333-3-1-S1.zip › bionlp_uima_repository-1.0.1/docs/api/src-html/edu/uchsc/ccp/util/nlp/annotation/AnnotationSet.html]

```
001    /*
002     *
003     * The Original Code is UCHSC Utilities.
004     *
005     * The Initial Developer of the Original Code is University of Colorado.  
006     * Copyright (C) 2005.  All Rights Reserved.
007     *
008     * UCHSC Utilities was developed by the Center for Computational Pharmacology
009     * (http://compbio.uchsc.edu) at the University of Colorado Health 
010     *  Sciences Center School of Medicine with support from the National 
011     *  Library of Medicine.  
012     *
013     *
014     * Contributor(s):
015     *   William A. Baumgartner <william.baumgartner@uchsc.edu> (Original Author)
016     *   
017     * Created: January 2006
018     * Dependencies:
019     * TODO:   
020     * Changes:
021     * Notes: 
022     */
023    package edu.uchsc.ccp.util.nlp.annotation;
024    
025    import java.io.PrintStream;
026    
027    public class AnnotationSet implements Comparable {
028        private final boolean DEBUG = false;
029    
030        private String annotationSetName;
031    
032        private String annotationSetDescription;
033    
034        private Integer annotationSetID;
035    
036        public AnnotationSet(Integer annotationSetID, String annotationSetName, String annotationSetDescription) {
037            this.annotationSetID = annotationSetID;
038            this.annotationSetName = annotationSetName;
039            this.annotationSetDescription = annotationSetDescription;
040        }
041    
042        public String getAnnotationSetDescription() {
043            return annotationSetDescription;
044        }
045    
046        public void setAnnotationSetDescription(String annotationSetDescription) {
047            this.annotationSetDescription = annotationSetDescription;
048        }
049    
050        public Integer getAnnotationSetID() {
051            return annotationSetID;
052        }
053    
054        public void setAnnotationSetID(Integer annotationSetID) {
055            this.annotationSetID = annotationSetID;
056        }
057    
058        public String getAnnotationSetName() {
059            return annotationSetName;
060        }
061    
062        public void setAnnotationSetName(String annotationSetName) {
063            this.annotationSetName = annotationSetName;
064        }
065    
066        public String getStorageLine() {
067            return annotationSetID + "|" + annotationSetName + "|" + annotationSetDescription;
068        }
069        
070        public void printStorageLine(PrintStream ps) {
071            ps.println(getStorageLine());
072        }
073    
074        // compares on set name only. ID is not considered
075        public int compareTo(Object annotationSet) {
076            if (annotationSet instanceof AnnotationSet) {
077                String setName = ((AnnotationSet) annotationSet).getAnnotationSetName();
078    
079                if (DEBUG) {
080                    System.out.println("COMPARING ANNOTATION SETS:");
081                    ((AnnotationSet) annotationSet).printStorageLine(System.out);
082                    this.printStorageLine(System.out);
083                }
084    
085                if (this.annotationSetName.equalsIgnoreCase(setName)) {
086                    return 0;
087                } else {
088                    return -1;
089                }
090            } else {
091                return -1;
092            }
093        }
094    
095        @Override
096        public boolean equals(Object annotationSet) {
097            if (compareTo(annotationSet) == 0) {
098                return true;
099            } else {
100                return false;
101            }
102        }
103    
104        @Override
105        public int hashCode() {
106            String hashkey = annotationSetName;
107            if (hashkey != null) {
108            hashkey = hashkey.toLowerCase();
109            } else {
110                hashkey="defaultAnnotationSet";
111            }
112            return hashkey.hashCode();
113        }
114    
115    }
```
